# Subcutaneous Forehead Island Flap for Nasal Reconstruction

**Published:** 2012-05-30

**Authors:** A Ebrahimi, M H Kalantar Motamedi, N Nejadsarvari, E Shams Koushki

**Affiliations:** 1 Department of Plastic Surgery, Trauma Research Center, Baqiyatallah University of Medical Sciences, Tehran, Iran; 2 Department of Oral and Maxillofacial Surgery, Trauma Research Center, Baqiyatallah University of Medical Sciences, Tehran, Iran; 3 General Practitioner, Tehran University of Medical Sciences, Imam Khomeini Hospital, Tehran, Iran; 4 General Practitioner, Trauma Research Center, Baqiyatallah University of Medical Sciences, Tehran, Iran

**Keywords:** Surgical flaps, Esthetic, Forehead, Basal cell carcinoma, Reconstruction

## Abstract

**Background:**

Reconstruction of nasal skin after tumor resection is imperative for full patient rehabilitation; and use of similar skin is necessary to achieve best esthetic and functional results.

**Methods:**

This clinical series study represent management of patients with large nasal defects (up to 4x7 cm) using subcutaneous pedicle island paramedian forehead flap, during a period of 2007-2009, 8 patients with large nasal defects were repaired with this flap, among them 5 patients were male and 3 patients were female with mean age of 53 years, all cases were reconstructed with island pedicle flap in a single stage.

**Results:**

Good and satisfying results were achieved in all cases except for one case that was operated again for debulking of flap.

**Conclusion:**

Island paramedian forehead flap provides esthetic and functional results in a single stage reconstruction of defects with various sizes and locations. This variation of forehead flap is a good choice especially for those patients that have problems with cost or problem with multistage reconstruction of nasal defects.

## Introduction

The history of nasal reconstruction began in approximately 600 BC with Sushruta`s transposition of a cheek flap for nasal reconstruction.[1] Hakim Dina Nath Kanghiara claimed that his ancestors used forehead flaps for nasal reconstruction in Kangara, India beginning about AD1440.[2]

The forehead is acknowledged to be one of the best, if not the best, donor sites for reconstruction of postoperative nasal defects after ablation in cancer patients. The versatility, color match, and texture are among the benefits of this flap. However, it has two major disadvantages including (i) It is stiff, flat, and thicker than normal nasal skin, and thus molding from a 2-dimensional to a 3-dimensional shape is difficult, and (ii) There is a donor-site defect that requires coverage using a split-thickness skin graft.[3]

Soft tissue repair of skin defects in the middle of the face is very important; and attention to its functional and aesthetic outcome toward successful rehabilitation of the patient should be concerned. In the majority of these cases, soft tissue reconstruction using local and regional flaps is indicated after choosing an appropriate treatment plan based on location, size of the defect, the patient's age and his or her wishes. Flaps from the nasal skin, glabella, forehead, as well as nasolabial fold, provide good possibilities to cover the defect and ensure the existence of equivalent color and texture.[4]

## Materials and Methods

This is a prospective case series study from 2007 to 2009, 8 patients with basal cell carcinoma of nasal skin was treated with the present method. The patients were 45 to 66 years old with mean age of 53 years. There were three female and five male patients. The size of the island flap was 4×2 to 7× 5 cm according to nasal defects. The island flaps were transposed subcutaneously while 180 degree rotation was used in all cases. The nutrient vessel in all flaps was supratrochlear artery. The average follow up time was 15 months (3-24 months), and no recurrence occurred. All patients were non-smoker and non-diabetic.

**Fig. 1:**
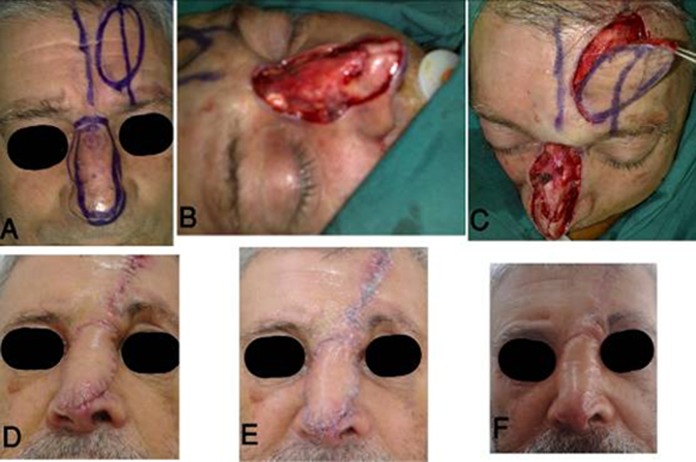
Subcutaneous pedicle island paramedian forehead flap for large nasal skin reconstruction. (A) Multiple nasal BCC and design of tumor excision and flap for reconstruction, (B) Tumor was excised, (C) Release of the forehead flap, (D) Early post-operation (E), One month post-operation (F) and three months post-operation.

In this type of forehead flap after excision of nasal skin tumor, design of the paramedian forehead flap based on supratrochlear vessels was done (Figure 1). It is necessary to design location of the island flap; so that flap pedicle can easily rotate 180 degrees subcutaneously and reaches to the nasal defect without tension. Furthermore, we must determine the correct location of pedicle with Doppler probe and should determine design of skin island along this artery; then release and preparation of the flap from distal to proximal was done. Deep dissection of the flap must be supraperiosteal (but in proximal part of the flap); to release the tunneled pedicle, we must dissect in two planes (subcutaneous and subperiosteal, 2-3 cm) toward the supraorbital rim; and width of the pedicle was approximately 1 cm. Pedicle contains vessels, forehead muscle and underlying periosteum that comprised no limitation in rotation of the flap. For preparation of the subcutaneous tunnel in the subcutaneous plane, dissection was done from supraorbital rim toward nasal defect; the width of a tunnel must be two folds of flap pedicle and with no tension. In addition, we excised procerous muscle for reducing swelling in the tunnel portion of the flap. There was no skin graft necessity in the donor site of the island paramedian forehead flap.

After operation, we had some swelling and venous congestion in the subcutaneous plane in the glabella but with a period of time, the swelling reduced; also distance between two eyebrows preserved without any unusualness in contour or location of eyebrows.

## Results

**Fig. 2:**
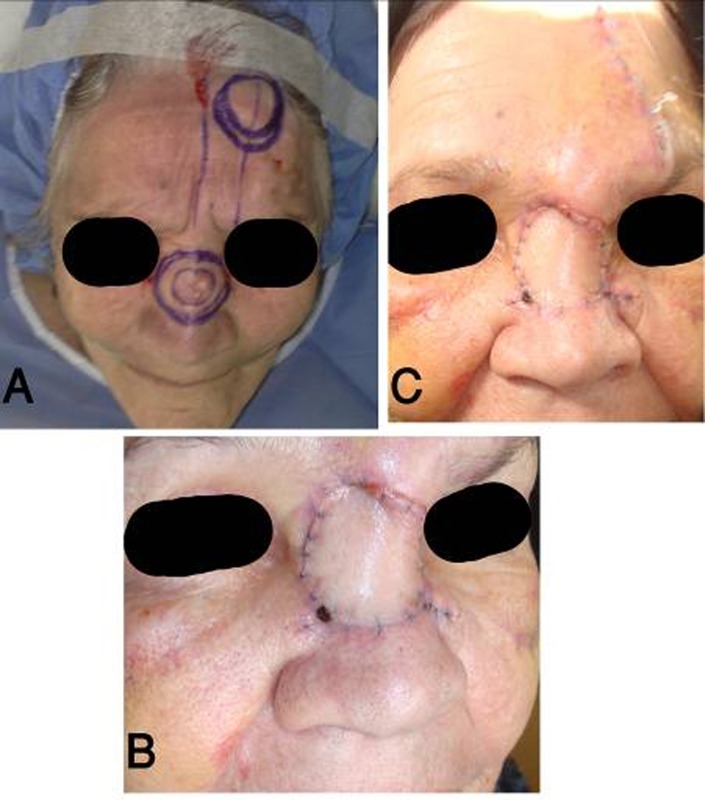
Basal cell carcinoma of the nasal dorsum: (A) Design of the island forehead flap based on supratrochlear artery, (B) Two weeks post-operation and (C) One month post-operation: reducing of glabellar swelling.

Island paramedian forehead flap can be used for reconstruction of large nasal skin defects in the zone III (zone I: upper dorsum, zone II: lower dorsum above the nasal tip); results of reconstruction with this flap were superior to skin graft or pedicle forehead or nasolabial flaps. Furthermore, advantages of this flap were the single-stage procedure without needs to revision and preservation of inter-brows distance. Some swelling of the glabella was reduced with a period of time and was acceptable. Donor site defect was repaired primarily with releasing of forehead muscle. Distal end of the island flap could be extended to the hairline and direction of the island vertically or oblique was dependent on the direction of supratrochlear artery (by means of Doppler probe), and usually it was vertical (Figure 2). If a patient had a transverse incision in the forehead in the region of flap design, we were not able to use this kind of flap, and other options must be considered for nasal reconstruction.

## Discussion

The goals of nasal reconstruction are the restoration of an esthetic, functional nose with minimization of the donor site deformity.[5] Reconstruction of large nasal defects is a major issue for plastic surgeons, reconstruction with local flaps or skin grafts, has an advantage that it can be accomplished in one stage but in any reconstruction, we must consider esthetic results. Local flaps from the adjacent skin provide excellent color and texture match to avoid two stage operations and obtain better esthetic results, we devised single stage tunneled paramedian forehead flap for large nasal skin reconstruction.

As this flap was transferred as an island flap, scars left on the forehead consisted only of the circumference of the island. Furthermore, there was little dog-ear remaining on the forehead. Inter-brow distance and eyebrow position were preserved in this method. In addition, this type of nasal reconstruction was done in a single stage, and this was a low-cost operation for patients.

Okada and Maruyama reported a forehead flap based on the wide subcutaneous pedicle, including bilateral supraorbital and supratrochlear vessels, compared with them, the pedicle of the island flap was much longer and narrower about 1 cm wide, including only unilateral supratrochlear vessels.[6] We also included periosteum in pedicle for better vascularity (Deep periosteal branch of supratrochlear artery is in periosteum 11). Kilinic and Bilen reported one stage reconstruction island flap with a maximum size of 6×7 cm.[7]

Some authors reported a forehead flap, including galea for lower one third nasal reconstruction. They used this flap for large nasal full thickness defects, according for hundreds if not thousands of years, nasal defects were reconstructed using pedicle forehead flaps.[8][9][10] According to a study by Reece, supratrochler artery was the axial blood supply to the paramedian forehead flap. This study confirms that distal part of this flap to be random.[11] The forehead is said to support random flaps five times the length of the base.[12] Of course in patients who were smoker, in diabetic and patients with transverse forehead scars, this type of paramedian forehead flap was unsuitable and was at risk of necrosis. We dissected flap pedicle in a tunnel portion subperiosteally for capture of deep periosteal branch of supratrochlear artery. Periosteal branch of artery extended beyond 3 cm above the supraorbital rim and sends additional perforators into the flap.[11]

For nasal reconstruction, it is necessary to use skin compatible with the color and texture for nasal reconstruction. Flap obtained from adjacent were the most appropriate for this purpose.[13] The most preferred treatment in nasal skin cancer was a safe tumor removal with an acceptable functional esthetic result. Firat reported second primary cancer occurrences on forehead flap after reconstruction of nasal carcinoma.[14] For prevention of tumor recurrence, we excised tumor with safe margins at all sides. Single stage reconstruction has many advantages over a two or three-stage reconstruction, single stage repair avoids the disfiguring elephant trunk deformity of the external pedicle.[15] In tunneled forehead flap, interbrow distance was preserved and length of scar in the forehead was shorter than the pedicle flap, in addition it was a single-stage operation and did not need a wound care, and the patient could use eyeglasses earlier than the pedicle flap. Converse and wood Smith described single stage forehead flap, which was an island forehead flap base on a wide pedicle, the flap was tunneled under intact glabella skin, which led to significant venous congestion and fullness in the glabellar region.[16]

In a study that was done in nine consecutive patients with subtotal to supratotal nasal defects, (Quetz and Ambrosch, 2011), three-stage forehead flap combined with the septal pivot flap technique was used, seven cases were repaired completely. With this technique, results had significantly improved and were stable to date.[17] In a study, 48 reconstructions for nasal defects were performed using forehead flap technique. Nasal defects of the dorsum, alar, tip, columella, and septum were successfully treated. Graft take was successful in all patients.[3] To reduce the venous congestion, we used subperiosteal dissection in a tunneled portion of pedicle and diameter of the tunnel that was dissected two times of pedicle width, furthermore we used narrow pedicle less than 1.5 cm width and resection of procerous muscle, this changes all helped to minimize contour problem and glabella fullness.

The most significant advantage of this flap was the ability to bury the pedicle, obviate the second stage, preservation of inter brows distance and limited scar length in the forehead donor site. The patient can use eyeglass earlier and cost of operation was less than multiple stage operation. This variation of forehead flap was a good choice for nasal skin defects, especially for those patients that had problems with cost or multistage reconstruction of nasal defects.
